# Psychophysiological responses of shame in young children: A thermal imaging study

**DOI:** 10.1371/journal.pone.0290966

**Published:** 2023-10-09

**Authors:** Sho Ohigashi, Chifumi Sakata, Hika Kuroshima, Yusuke Moriguchi

**Affiliations:** Graduate School of Letters, Kyoto University, Yoshidahonmachi, Kyoto, Japan; University of Valencia: Universitat de Valencia, SPAIN

## Abstract

Shame can be defined as the emotional response to one’s violations of rules being exposed to others. However, it is difficult to objectively measure this concept. This study examined the psychophysiological indicators of shame in young children using behavioral methods and thermography, which measures facial temperatures that reflect blood flow changes related to emotions. Four- to six-year-old children participated in an “animal guessing game,” in which they lied about having violated a rule. They were assigned to either the exposure or the non-exposure group. In the exposure group, participants’ lies were exposed by the experimenter, whereas in the non-exposure group, their lies were not. Results showed that at the behavioral level, participants in the exposure group expressed characteristic behaviors of shame (e.g., embarrassed smiles) more often than those in the non-exposure group. Moreover, the nasal temperatures of participants in the exposure group were higher than those of participants in the other group after the lie was exposed. These results suggest that participants’ lies being exposed induced psychophysiological responses and consequently raised their nasal temperature. This finding indicates that psychophysiological responses can enable us to objectively measure higher-order emotions in young children.

## 1 Introduction

### 1.1 Definition of shame

People experience various emotions in their interactions with others and their environments. There are basic emotions such as happiness, sadness, and anger, and higher-order emotions such as shame, guilt, and embarrassment [1]. Lewis suggested that emotions develop in stages, from basic to higher-order, during infancy and early childhood [1]. In particular, 2- and 3- year-old children experience shame or guilt once their self-consciousness and self-evaluation skills enable them to assess themselves against social standards or rules [1].

Lewis proposed that children are likely to feel shame, guilt, or regret when they fail to meet established social standards [1]. Several theories are proposed to explain individuals’ experience of shame. For instance, exposure theory suggests that individuals may experience blushing when confidential information they wish to conceal becomes exposed or is threatened with exposure [2–4]. The current study is based on an attribution theory of shame and guilt. Tangney et al. [5]. propose that shame and guilt arise when one evaluates oneself negatively because of rule violations (e.g., lying, cheating, stealing)., a shamed person focuses on their entire selves, while a person experiencing guilt focuses on the negative effects of their behaviors on others. For example, children may feel guilty when they break a friend’s toy and the friend cries. Considering these definitions, this study defined shame as the emotional response to one’s voluntary rule violations being exposed. Further, we presumed that children are more likely to feel shame than guilt when their behaviors do not hurt others. It is necessary to investigate the behavioral and physiological expressions of shame, given its importance to emotional development during childhood [1, 5].

### 1.2 Psychophysiological indicators

Shame has been measured in existing studies using behavioral indices [6, 7]. For example, Witkower and Tracy [8] found that shame is behaviorally characterized by a collapsed upper body, which encompasses contraction of limbs, bowing of the trunk, and narrowing of the chest. Further, studies have found that children tend to avoid others when ashamed [5]. It is difficult to measure shame using only behavioral indices, so combining behavioral indices with physiological indices allows a more accurate measurement of shame.

Shame is a specific form of stress, which is a self-conscious emotion emerging from the internalization of social devaluations perceived from others [9]. Stress is intricately linked with fluctuations in skin temperature. Specifically, periods of heightened arousal, often triggered by acute stress, can increase blood flow, consequently elevating the temperature of adjacent areas [10]. Skin temperature is widely recognized as a reliable psychophysiological index for emotion assessment. Temperature is measured using thermography, which uses an infrared camera to detect heat patterns and blood flow in body tissues. The advantage of using thermography with children is that it is non-invasive, and hence both safe and useful for children who are too young to report their emotions in their own words. Cuevas et al. [11] reviewed studies that used thermography to measure temperature changes according to different emotions, and found that facial temperature changes reflect emotional responses such as fear or anxiety. Ioannou et al. [12] measured children’s emotional responses to guilt using thermography; they found that nasal temperatures decreased when children were caring for those whom they had hurt, which is a behavioral characteristic of guilt. These results are important, as they revealed that thermography can be used for basic and higher-order emotions. Nevertheless, to the best of our knowledge, few previous studies have examined shame using thermography.

In most studies that have used psychophysiological indices, other measures were used to improve validity. For example, Ioannou et al. [12] assessed children’s guilt using thermography and behavioral indices. Goulart et al. [13] measured children’s emotions using thermography and an emotional self-assessment scale. Moreover, people’s tendency to feel shame differs according to their personalities [14]. For instance, Abe [14] found that in adults, the tendency to feel shame was negatively correlated with extraversion. Therefore, in addition to using thermography, we recorded participants’ behaviors, asked participants to report their emotions subjectively, and assessed participants’ personalities.

### 1.3 The peeking paradigm

To induce shame in the current study, a situation was designed in which participants voluntarily violated a rule and lied about it, and their lies were exposed by the experimenter. The peeking paradigm, in which participants are instructed not to peek at toys when the experimenter is absent, has been used in previous studies on children’s lies [15–17]. Peeking during the experimenter’s absence is considered to be the rule violation. Previous studies using this paradigm have shown that young children tend to violate the rule and conceal their violation when experimenters inquire about it. Concealing their behavior indicates that they understand the standard that they should obey the rule. Therefore, this paradigm is useful to induce shame.

### 1.4 Research question

This study aimed to measure psychophysiological indicators of shame in young children using behavioral indices and thermography. When people feel ashamed, they blush and, the nose has a high concentration of peripheral vessels [18]. Therefore, this study predicted that the participants who had felt ashamed would have an elevated nose temperature. Moreover, we expected that they would score higher on personality traits linked to the tendency to feel shame.

## 2 Method

### 2.1 Participants

We recruited participants between 4 and 6 years of age. By that age, they are old enough to lie to hide their transgressions [19], self-evaluate, and feel shame [1]. Participants included 54 children (*M* = 66.8 months, *SD* = 6.7 months, age range = 54–81 months, males = 27), 45 of whom (*M* = 67.6 months, *SD* = 6.7 months, age range = 54–81 months, males = 26) were included in the final analyses. One of the excluded participants did not fit in any of the groups (see Section 2.3.1), whereas others were unable to complete the task due to technical problems or procedural mistakes.

The experimental design in the present study was similar to the study by Ioannou et al. [12], in which they used thermography to measure higher-order emotions. Ioannou et al. [12] included 15 participants; hence, we assigned 15 participants to each of our three conditions. The participants were registered with the Kyoto University Baby Researcher Program or sent by Cross Marketing Co. They lived in the Kinki region of Japan. Ethical approval was granted by the local ethics committee of Kyoto, Japan (2-P-8). All procedures performed in this study complied with the ethical principles of the Declaration of Helsinki regarding research with human participants. Written informed consent was obtained from the parents of all participants (children, in this case) involved in the study.

### 2.2 Materials

#### 2.2.1 Task materials

A stuffed dog, chicken, rabbit, and seal were used, and their sounds were sourced from the Internet and played on the experimenter’s smartphone or laptop.

#### 2.2.2 Thermography

A FLIR T650sc thermal imaging camera with an image frequency of 30 Hz, thermal sensitivity of less than 20mK, infrared resolution of 640*480-pixel, and spectral range from 7.5 to 14μm was used for thermography. It was connected to a Surface Pro 6 LQK-00014 tablet, which also recorded behavioral data using Debut Professional ver. 6.15 video capture software. The thermal imagining camera was placed in a corner of the laboratory, with participants being recorded from a distance of approximately 2 meters.

#### 2.2.3 Facial expression scale

**Fig 1:**
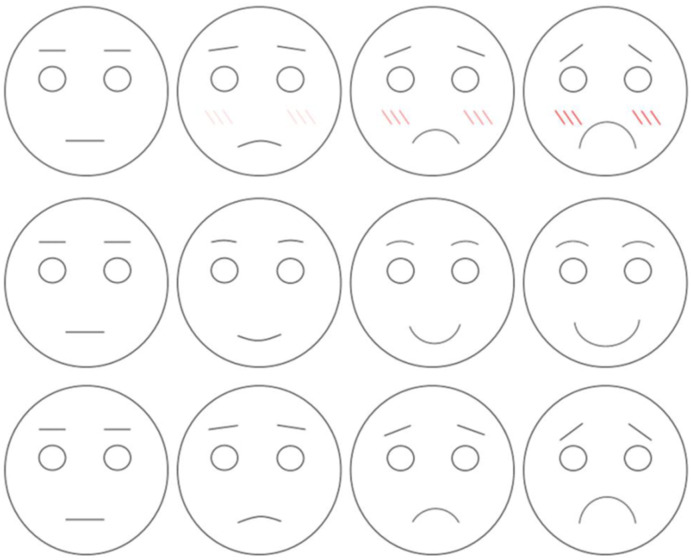
Facial expressions for emotions (from top to bottom: Shame, happiness, and sadness). Each expression was scored on a four-point scale (e.g., 1 = not ashamed at all, 2 = slightly ashamed, 3 = ashamed, 4 = very ashamed).

We created three four-point facial expression scales for shame, sadness, and happiness (Fig 1), based on the study by Gautam et al. [20].

#### 2.2.4 Temperament scale

We used the shyness scale from the first edition of the Children’s Behavior Questionnaire (CBQ) [21], in which shyness is defined as a slow or inhibited approach toward novel or uncertain situations. This scale consists of 13 items (e.g., “Is sometimes shy even around people s/he has known a long time”). Shyness was used as a temperamental indicator, as it is associated with proneness to experience shame [22]. While the experimenter was caring for the participants, their parents answered the shyness scale from the CBQ, on which items are rated on a seven-point scale (1 = not true at all, 7 = exactly true). If there was no situation like the one described in any item, X (none) was selected. The Cronbach’s α for this scale was .92 in this study.

### 2.3 Procedure

We controlled for environmental factors that have been shown to influence temperature measurements [11]. The experimenter kept the room temperature and humidity in the laboratory constant (room temperature: *M* = 24.2°C, *SD* = 1.5°C; humidity: *M* = 42.8%, *SD* = 13.4%). To acclimatize their body to the room temperature, the participants spent approximately 30 minutes in the laboratory, during which they used personal computers at designated desks to perform a cognitive task with their parents. After the cognitive task, they took a five-minute break while remaining seated, after which they started the main task. Since facial temperatures were being observed, the experimenter used hair clips to hold back any hair that was obstructing a clear view of participants’ foreheads.

#### 2.3.1 The main task: Animal guessing game

**Fig 2:**
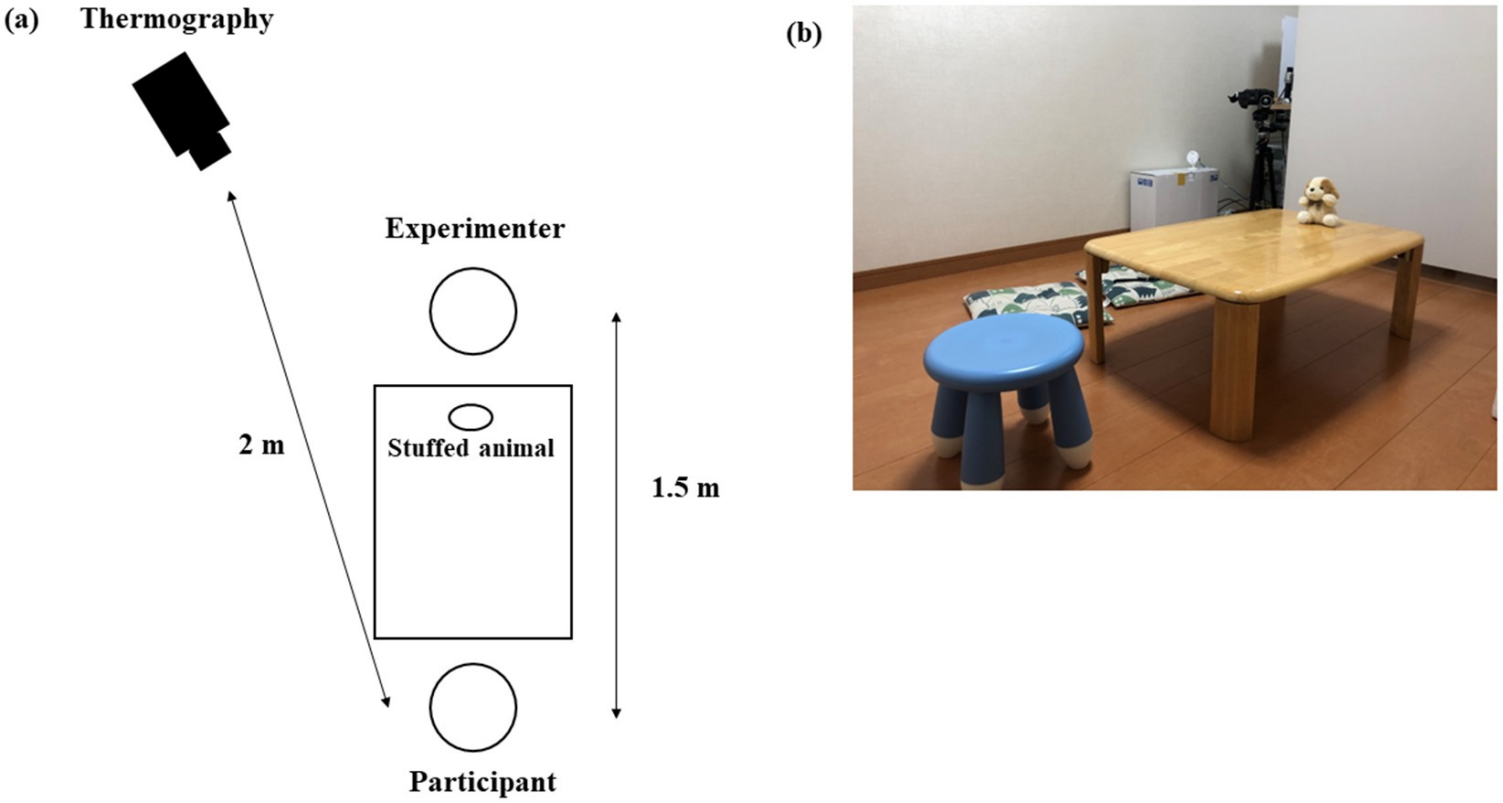
(a) A scheme of the laboratory. (b) The laboratory. The participants sat on the round chair in the foreground of the image.

Participants completed the animal guessing game based on the peeking paradigm [17] without their parents. In this game, participants guessed the correct animal based on the sounds presented. First, they practiced guessing while sitting in a circle facing the experimenter (Fig 2), after which the testing phase commenced.

The practice phase consisted of two trials: dog sounds for the first, and chicken sounds for the second, both of which the pilot study have shown to be easily recognizable for young children. In the first trial, participants were told to turn their backs to the experimenter and not turn around until the experimenter instructed them to do so. After checking that the participants had turned their backs, the experimenter placed the stuffed animal on the front desk, played the relevant sounds on a smartphone or laptop, and asked participants to identify the correct animal. After answering, participants were allowed to face the experimenter and check which stuffed animal was on the desk. Then, they were told to turn around for the second trial, which was conducted similarly.

In the test phase, the experimenter told participants to turn around, and then placed a stuffed animal (either a rabbit or a seal) on the desk. Unlike in the practice phase, sounds unrelated to the animals were played. Immediately after playing these sounds, the experimenter played the ring tone of a telephone and told participants that she would leave the room to answer the phone. Before the experimenter left, the participants were instructed not to turn around to check which stuffed animal was on the desk. Participants remained alone after the experimenter left, but they were observed from an outside monitor for one minute. Participants who did not turn around were included in the no-violation group, and those who turned around were randomly assigned to either the non-exposure or exposure groups. Upon returning to the laboratory, the experimenter asked all participants whether they had turned around while they had been left alone (this was labeled as the confirmation question). Afterward, the experimenter asked different questions for each group:

***No violation group*:** Participants were asked whether the animal guessing game was difficult (the control question).
***Non-exposure group*:** The control question was asked.
***Exposure group*:** The experimenter told participants that they had been observed turning around through the hidden camera, and asked again whether they had turned around (the experiment question).

To induce shame, it was necessary to instigate negative self-evaluations by revealing the violation. Therefore, in our experimental exposure condition, experimenters asked the experimental question. The exposure group was expected to experience shame. A previous study [23] using the same paradigm reported that 71% of 5-year-old participants violated the rule. The ratio was similar in other studies [15–17] using this paradigm. Therefore, we expected that one third of the participants would not violate the rule. One participant honestly reported that he had turned around. He was asked the control question and excluded from subsequent analyses.

#### 2.3.2 Subjective reports of emotional experiences

After the test phase, participants reported the emotions they had experienced while being asked the control or experiment question. Each participant pointed to values from three different facial expression scales (Fig 1: shame, happiness, sadness) which were recorded by the experimenter.

#### 2.3.3 Soothing

After the game, the experimenter played together with the participants for approximately five minutes to sooth them, based on a previous studies using the same paradigm (e.g., [17]). Afterwards, when the experimenter or participants’ parents thought that participants may be stressed, *Eine Kleine Nachtmusik* was played for five minutes as supplementary care, since it has been found to induce positive emotions [24].

### 2.4 Analysis

**Fig 3:**
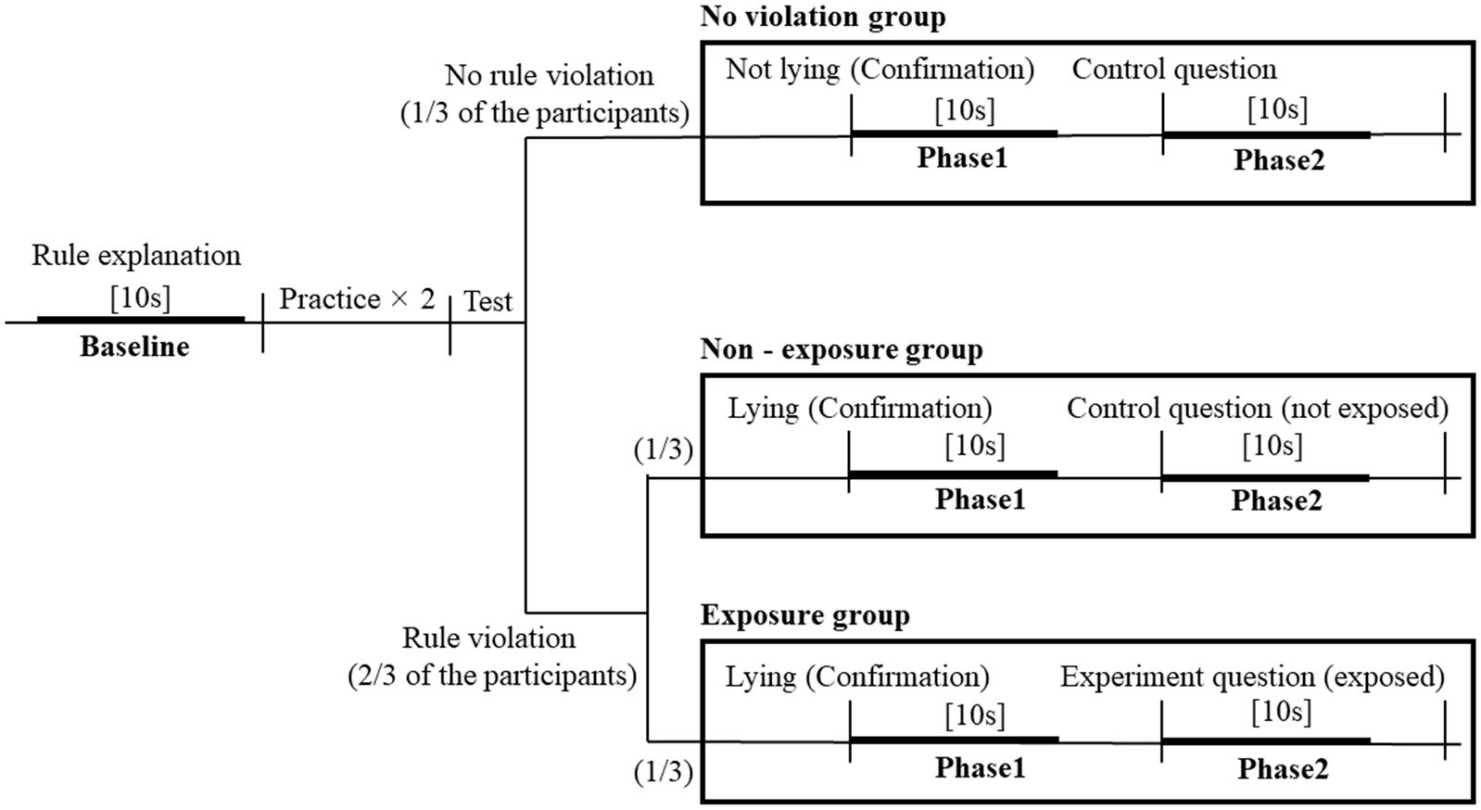
The flow of the animal guessing game. The thick lines represent the phases used in the analysis. Short vertical lines indicate changes in the procedure.

R (ver. 4.0.3) was used for subsequent analyses [25]. We divided the video data of all groups into two phases: Phase 1 was 10 s after the confirmation question, during which participants from the non-exposure and exposure groups lied about their rule violation; Phase 2 was 10 s thereafter, during which participants’ rule violation was exposed. In all groups, the test phase ended 30 s after the control or experiment question, regardless of whether the participants responded or remained silent. To set the baseline for temperatures, we used 10 s of video data during which the rules of the game were explained (Fig 3).

#### 2.4.1 Behavior data

To measure participants’ emotional states, two coders rated their behaviors during the animal guessing game in each phase. All coding was conducted based on the video data. This study used five behavioral indices (Table 1) based on previous studies that have measured behavioral expressions of shame [26–28]. For each behavior, coders rated whether each participant expressed it in any of the phases (0 = the behavior did not occur; 1 = the behavior did occur). One of the coders was aware of the hypothesis, while the other was not. The simple percentage agreements between coders ranged from .48 to .89. This study used simple agreement percentages and did not use the Kappa coefficient, which is commonly used to evaluate the interrater reliability of behavior coding. This is due to the nature of the kappa coefficients. Higher-order emotions, including shame, are difficult to identify in facial and behavioral expressions, and thus require precise definitions to code [8]. Due to the strict definitions of behaviors, the percentage of 0 (i.e., the behavior did not occur) in our coding was high. In this case, the value of Kappa decreased [29].

**Table 1:** Behavioral indices and their definitions.

Behavioral Indices	Definitions of the Behaviors
Bodily Avoidance (BA)	The body was moving during the experimenter’s question, and it became still and rigid after the question.
Bodily Tension (BT)	After the experimenter spoke, participants shrugged their shoulders and hung their heads.
Embarrassed Smile (ES)	Only the muscles around the mouth moved in response to the experimenter’s question, and the muscles around the eyes did not move: Non-Duchenne Smile.
Gaze Aversion (GA)	After the experimenter’s question, participants looked at the experimenter and immediately looked away.
Verbal Uncertainty (VU)	Participants stammered or became silent in response to the experimenter’s question.

To assess participants’ emotional states, we calculated the percentage of participants who expressed behavioral indicators of shame in each phase. A generalized linear mixed model assuming a binomial distribution was fitted to the proportion of participants who expressed each behavioral indicator; this proportion was considered the response variable and the group, phase, group/phase interactions, and random effects (behavioral indicators) were the explanatory variables. Tukey’s multiple comparisons was conducted as a post hoc test.

#### 2.4.2 Temperature data

The software FLIR ResearchIR Max (ver. 4.40.9.30) was used to measure temperature data from the thermal videos. Based on previous research [11], a rectangular region of interest (ROI) was set for the forehead and nose. Temperature data were analyzed for each ROI because temperature differences between facial parts were not relevant to this study. The ROI sizes were fixed for each participant. Merla and Romani [30], who measured facial temperatures using thermography, showed that temperature changes within 3–4 s. Therefore, this study recorded facial temperatures at approximately every 100 frames (3–4 s) in each phase.

As Cuevas et al. [11] pointed out, blood flow differs among individuals. Therefore, we standardized each participant’s temperature data from baseline to Phase 2, with a mean of zero and a standard deviation of one on R [25]. To measure temperature changes, we calculated the means of the standard score differences of temperatures between phases for each group. The Shapiro-Wilk test indicated that the standard score differences were not normally distributed. Therefore, we conducted the Kruskal-Wallis test to measure standard score differences between participants for both facial ROIs. The Mann-Whitney U test was conducted as a post hoc test. Bonferroni’s correction was used (alpha level = .025). This study conducted exploratory analyses on the eye and cheeks (S1 Fig), and determined correlations between behavioral and thermal data (S2 Fig).

## 3 Results

All relevant data are available on https://doi.org/10.6084/m9.figshare.23593143 and https://doi.org/10.6084/m9.figshare.20362611.v1.

### 3.1 Behavior analysis

Due to the problem with the connection between the thermography and the video capture software, behavioral video data was missing for some participants. Therefore, in behavioral analysis, 12 participants’ data was in no violation group and non-exposure group, and 13 in exposure group.

**Fig 4:**
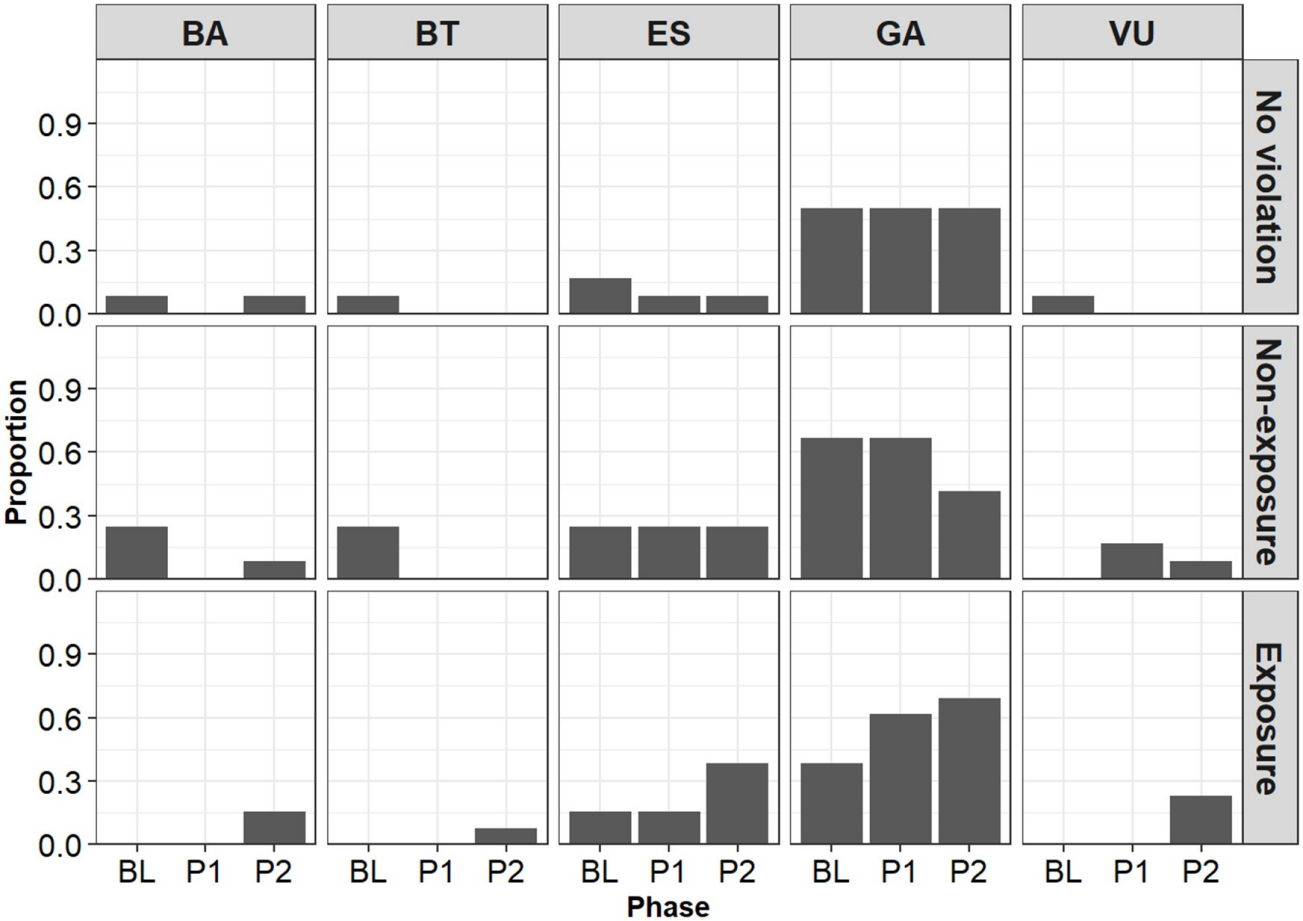
The proportion of the participants who expressed behavioral indices of shame in each phase. The vertical axis represents the proportion. The horizontal axis represents the phase. BL, P1, and P2 on the horizontal axis represent baseline, Phase 1, and Phase 2, respectively. For descriptions of each behavioral indicator (e.g., BA), see Table 1.

Fig 4 shows the percentage of participants who expressed behavioral indicators of shame in each phase. A generalized linear mixed model was fitted to the proportion of participants who expressed each behavioral indicator. The results showed significant interactions between groups and phases, *χ*^2^(4) = 14.15, *p* < .01. The results of simple main effect tests showed a significant simple main effect of group in Phase 2, *χ*^2^(2) = 7.79, *p* = .02. The results of Tukey’s multiple comparisons showed that in Phase 2, the proportion of participants who expressed behavioral indicators was significantly higher in the exposure group than in the no-violation group (*z* = 2.55, *p* = .03). The mean proportion was higher in the exposure group than in the non-exposure group, but the difference was not significant (*z* = 2.05, *p* = .10).

### 3.2 Temperature analysis

**Fig 5:**
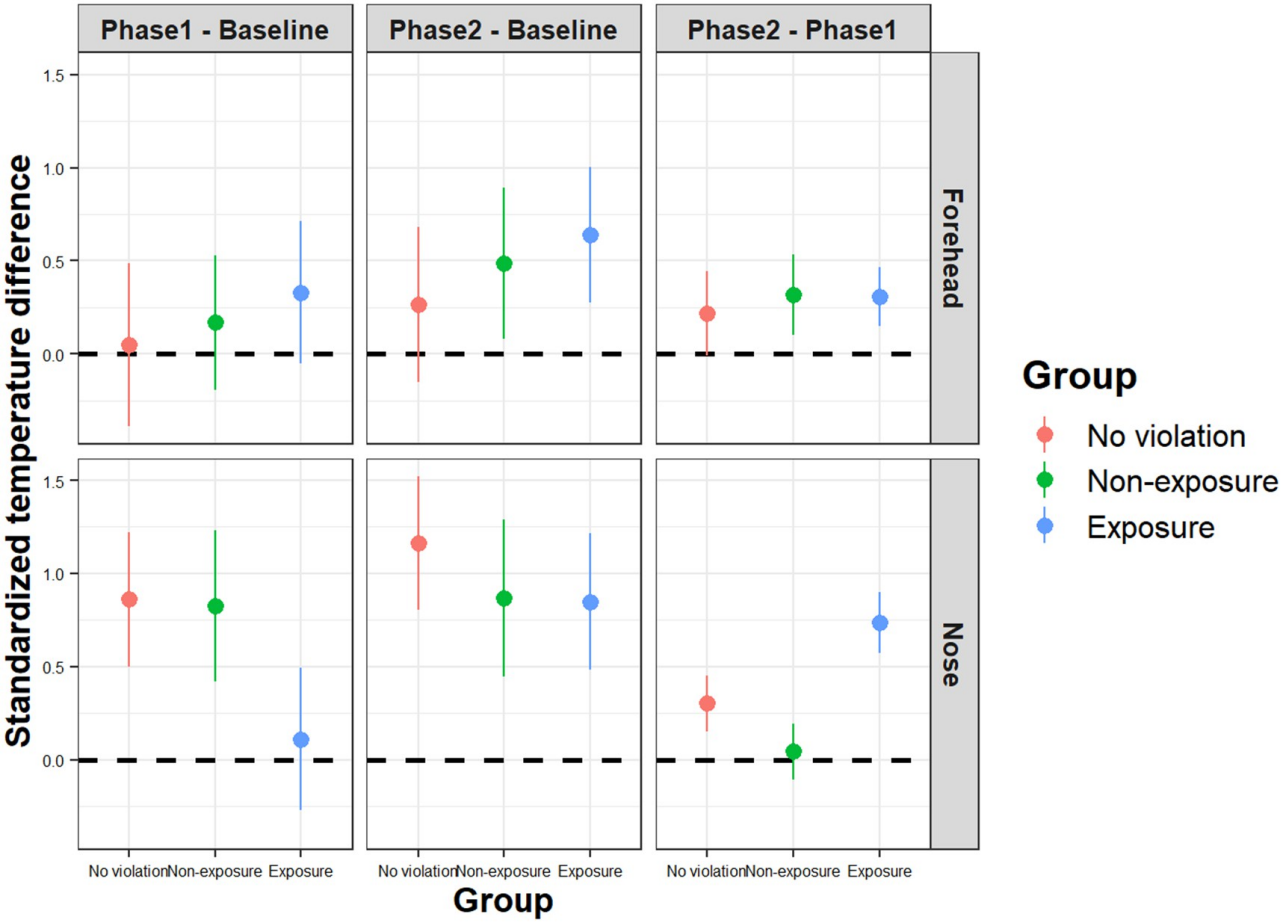
Means of standard score differences between phases for each group. Each dot represents the mean of standardized temperature change in each group. Error bars represent the standard error of the means.

**Fig 6:**
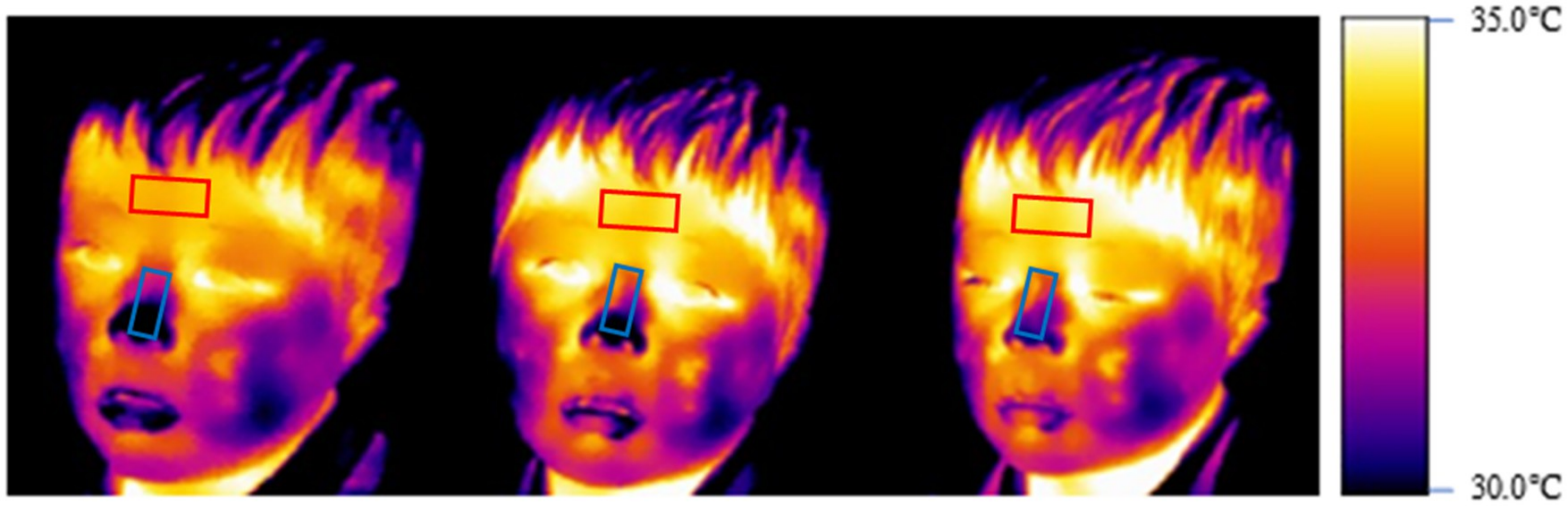
A thermal imaging example of the facial temperature changes from the baseline to Phase 2 in the exposure group. The first frame, second frame, and third frame show the temperature at baseline, Phase 1, and Phase 2, respectively. Color changes from darker to lighter shades signify a rise in temperature. The red region of interest is on the forehead and the blue region of interest is on the nose.

Fig 5 shows the means of the standard score differences of temperatures between phases for each group. Fig 6 illustrates a thermal imaging example of facial temperature changes from baseline to Phase 2 in the exposure group.

The Kruskal-Wallis test revealed that between Phase 1 and Phase 2, there was a significant effect of group on nose temperatures, *χ*^2^(2) = 8.83, *p* = .012, but not on forehead temperatures, *χ*^2^(2) = 1.40, *p* = .50. The Mann-Whitney U test showed that the standard score difference of the exposure group was significantly larger than that of the non-exposure group (*Z* = -2.82, *p* < .01, *r* = .75), and it was larger, but not significantly so, than the no violation group (*Z* = -2.05, *p* = .040, *r* = .53). From baseline to Phase 1, the standard score differences were not significant across all groups, nose: *χ*^2^(2) = 2.47, *p* = .29; forehead: *χ*^2^(2) = 0.01, *p* = 1.0.

### 3.3 Subjective reports

We calculated the mean scores of subjective reports for each group (Table 2). Results of analysis of variance (ANOVA) tests showed no significant differences for each emotion between groups, *F*(2, 39) = 0.17, *p* = .89.

**Table 2:** Mean scores and standard deviation for each emotion.

Group	Happiness		Sadness		Shame	
	*Mean*	*SD*	*Mean*	*SD*	*Mean*	*SD*
**No violation**	2.8	1.08	1.27	0.59	1.67	0.72
**Non-exposure**	2.8	1.37	1.4	0.91	1.67	0.90
**Exposure**	3.0	1.04	1.08	0.29	1.5	0.67

### 3.4 Temperament scale

The scores of the CBQ are presented in Table 3. ANOVA showed no significant difference in scores between groups, *F*(2, 42) = 0.43, *p* = .65. We also tested the relationship between shyness scores and nasal temperature differences in the exposure group but did not find any significant correlations (*r* = .28, *p* = .34).

**Table 3:** Mean shyness scores and standard deviation in each group.

	No violation group		Non-exposure group		Exposure group	
	*Mean*	*SD*	*Mean*	*SD*	*Mean*	*SD*
**Score**	4.3	0.9	4.0	1.0	4.4	1.3

## 4 Discussion

This study used behavioral observations and thermography to understand the relationship between facial temperature and shame among young children. The behavioral findings suggest that the exposure group felt shame. The non-exposure group also showed some behavioral expressions of shame, which may be because they may have felt negative emotions due to unexposed lying and therefore displayed behaviors associated with shame. Specifically, embarrassed smile was higher for Phase 2 compared with Phase 1 and baseline in the exposure group. This can be considered a form of appeasement [31]. Moreover, gaze aversion was lower in Phase 2 than in Phase 1 and baseline in the non-exposure group. The children could have been looking for cues to doubt whether the experimenter knew the truth.

Temperature analysis revealed that nasal temperatures among the exposure group increased from baseline to Phase 2, which is consistent with our prediction. Based on Giannakakis et al. [32], it is suggested that shame, a kind of stress induced high arousal and thus increased blood flow. It should be noted that these changes are not due to the act of lying, as temperature changes between baseline and Phase 1 were not significant. These results, coupled with the behavioral results, imply that the participants in the exposure group felt shame, which was reflected by increased their nasal temperatures. Fig 6 appears to display a noticeable contrast in lip color, which may be attributable to the experience of shame. However, given the nature of the animal guessing game wherein participants were required to respond to the experimenter, we cannot deny the possibility that the observed change in lip temperature may be a result of mouth movements associated with talking. Future research should be done to assess the point.

There were no significant differences between the subjective reports from groups, perhaps because participants, who were aged between 4–6 years, found it difficult to associate emotions they had experienced with corresponding metrics. This is supported by a previous study by Widen and Russell [33] who required 4- to 10-year-olds to label emotions by facial expressions or stories of cause and consequences, and found that stories were stronger cues than faces, especially for social emotions such as shame.

There were no significant differences in CBQ score between groups and no significant correlations between CBQ scores and temperature changes. This may be because CBQ scores were reported by participants’ parents, which may not accurately capture participants’ propensity to shyness. Overall, these findings suggest that a combination of psychophysiological and behavioral measurements can reveal complicated negative emotions that are likely to be missed in children’s verbal reports or their parents’ observations.

This study findings indicate that since thermal measurement can be applied to children, the development of higher-order emotions among children can be physiologically investigated. Further, thermography can assist in physiologically distinguishing between emotions that are difficult to distinguish according to behavior alone; for instance, our findings indicate that shame raises nasal temperatures; while guilt has been found to lower nasal temperature [12].

This study had some limitations. First, the animal guessing game is one of many methods to induce shame in children. It is important to check whether nasal temperatures rise in other shame-inducing situations. Second, factors other than emotions may have influenced nasal and forehead temperatures. For example, as Cuevas et al. [11] noted, metabolic rate or circadian rhythms can affect temperature changes. Experiments controlling for these factors may be able to measure emotions with greater accuracy. Third, the duration of each phase in our study could be considered short. Blushing is a well-documented physiological response to feelings of shame. Prior research has suggested that peak temperature changes associated with blushing tend to occur approximately 90 seconds after the onset of the blush-inducing situation [34]. Thus, extended phase durations might have facilitated the detection of temperature fluctuations attributable to blushing. Nevertheless, Merla and Romani [30] were able to detect temperature changes within a timeframe of 3–4 seconds, interpreting these as indications of arousal shifts. Consequently, it is plausible that our study measured temperature changes linked primarily to arousal, triggered by social stress, rather than those specifically associated with blushing.

This study analyzed correlations between behavioral and thermal data. However, there were no significant results (see S2 Fig), so cross-validating is the next important step to ground physiological mechanisms of shame. Other previous studies such as that by Merla and Romani [30] have shown that temperature changes in thermal data corresponded to emotional sweating.

In conclusion, our findings suggest that when young children were exposed to lies, they experienced psychophysiological responses that increased their nasal temperature. This research suggests that higher-order emotions in young children can be objectively measured through psychophysiological responses.

## Supporting information

S1 Fig Means of standard score differences between phases for each group. (TIF)

S2 Fig Correlation between the temperature and the behavior data in each indicator. (TIF)

S1 File Supporting information. (DOCX)
