# Supplementary figures and images for: Psychophysiological responses of shame in young children: A thermal imaging study
Source: PLoS One. 2023 Oct 9;18(10):e0290966. doi: 10.1371/journal.pone.0290966 (PMC10561869; doi:10.1371/journal.pone.0290966)

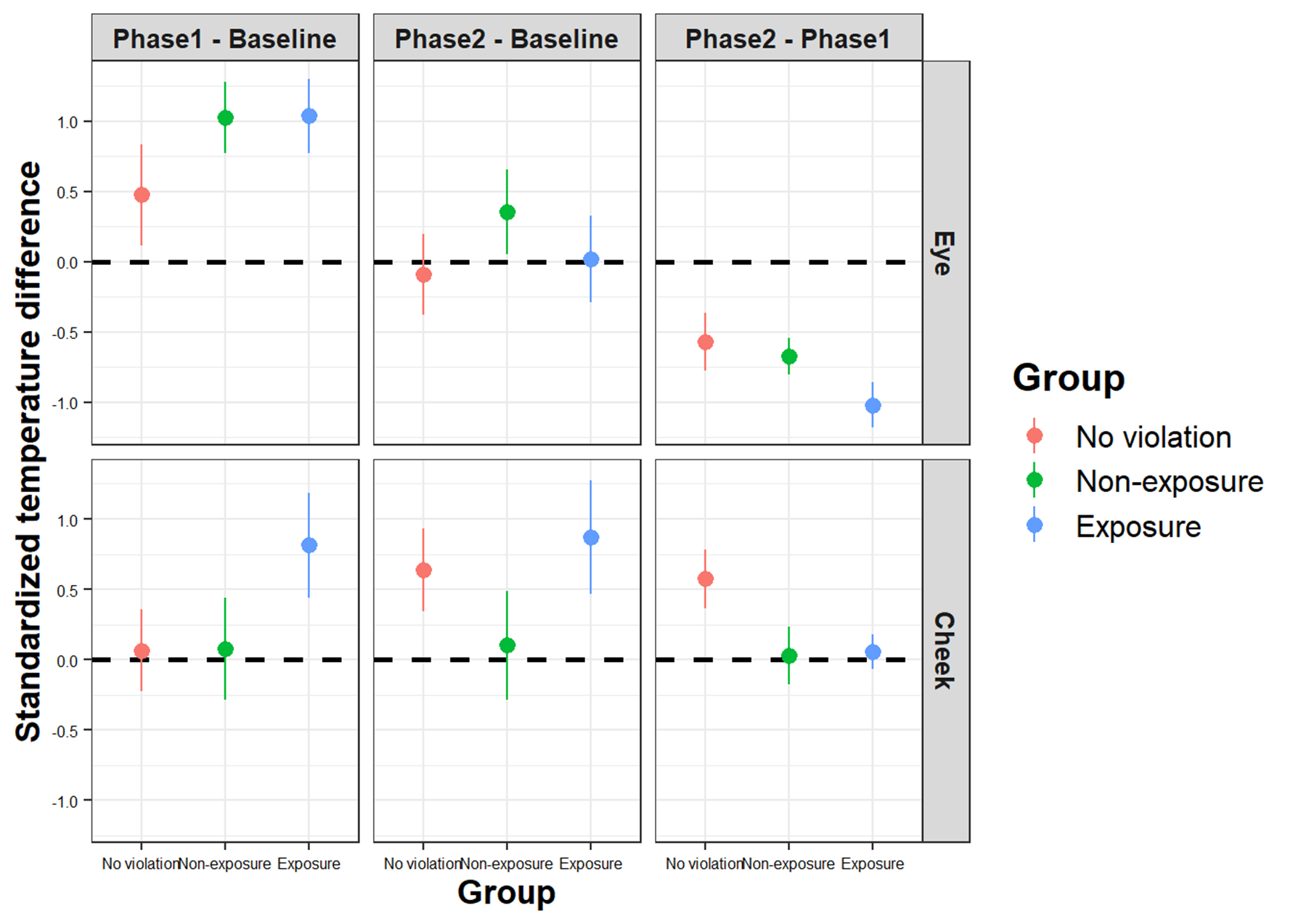

Supplement: S1 Fig — (TIF) [file pone.0290966.s001.tif]

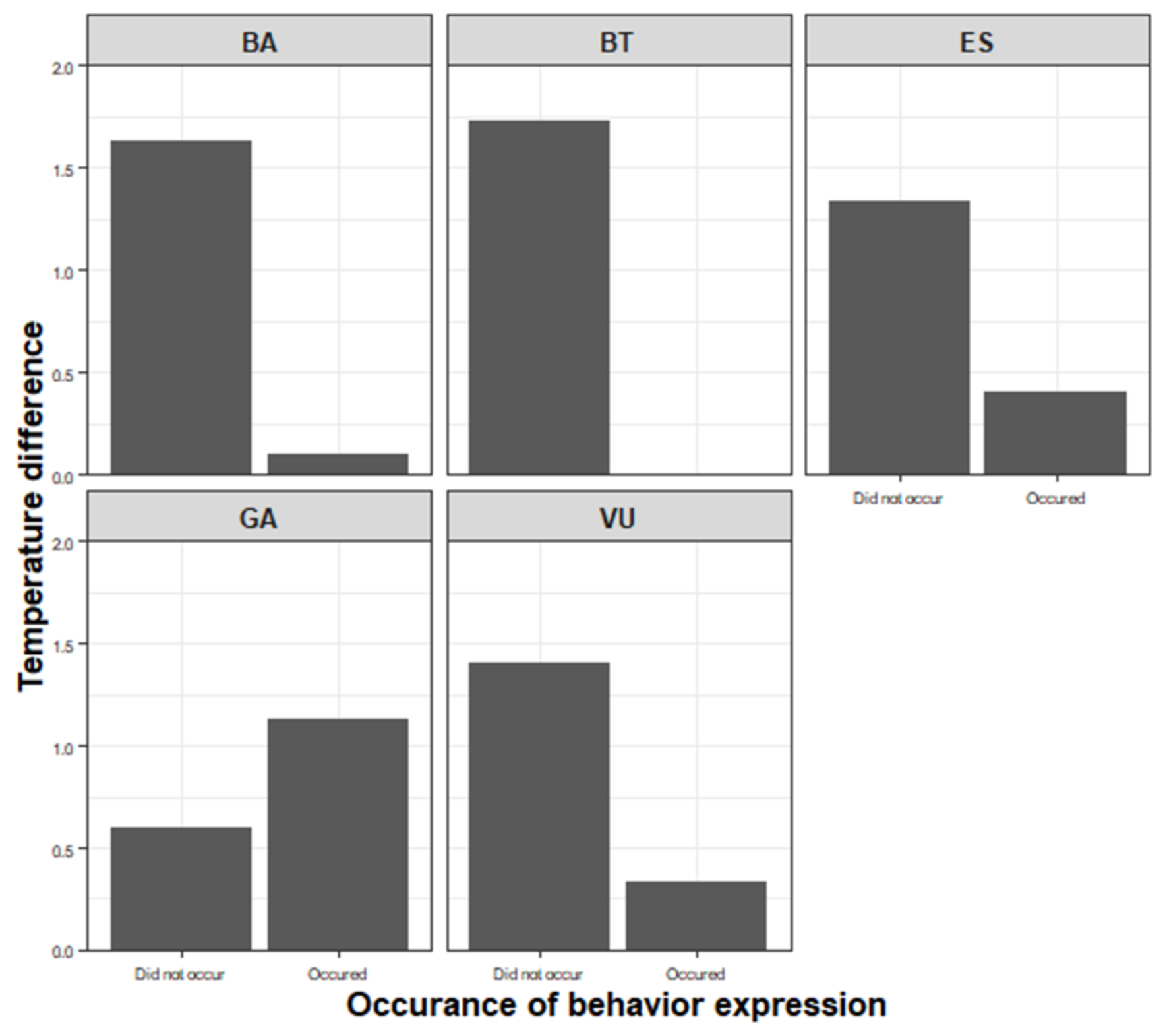

Supplement: S2 Fig — (TIF) [file pone.0290966.s002.tif]
