# Supplementary material for: Psychophysiological responses of shame in young children: A thermal imaging study
Source: PLoS One. 2023 Oct 9;18(10):e0290966. doi: 10.1371/journal.pone.0290966 (PMC10561869; doi:10.1371/journal.pone.0290966)
Supplement: S1 File — (DOCX) [file pone.0290966.s003.docx]

**Supporting information**

Although many of the studies tested the temperature of the forehead and the nose, some previous study focused on the eye and the cheek to measure emotion using thermography [8]. Therefore, this study conducted exploratory analyses on the eye and cheeks. S1 Fig shows the means of the standard score differences between phases for each group.

　　The Kruskal-Wallis test revealed that between phase1 and phase2, there was no significant effect of participants’ groups on the cheek standardized temperatures (*χ*²(2) = 4.44, *p* = .11). From baseline to phase1, the standard score differences were not significant on the eye (*χ*²(2) = 1.57, *p* = .46).

　　It is suggested that the temperature of the cheeks did not change because the blood around the musculature of the cheek is connected to the one of the eyes [32]. According to the definition of the embarrassed smile (Table 1), the muscles around the eyes did not move. Therefore, this may be why the temperature on the eye and the cheek did not change.

To validate the temperature data, it is important to correlate these data and another data. Therefore, this study analyzed the relation between behavioral and thermal data (S2 Fig). The indicators are Bodily Avoidance (BA), Bodily Tension (BT), Embarrassed Smile (ES), Gaze Aversion (GA), and Verbal Uncertainty (VU). See Table 1 for details about each behavior indicator.

　　A generalized linear mixed model assuming a binomial distribution was fitted to the occurrence of behavior expression; this was the response variable and the temperature differences, and random effects (behavioral indicators), were the explanatory variables. The results showed significant effect (*χ*²(1) = 2.74, *p* = .1). It may be because the facial temperature changes do not always reflect the influence of the behavioral indicators. For example, in the experiment where the participants are seated, their facial temperatures are reported to change [8].
